# Tumor Infiltrating Lymphocytes Signature as a New Pan-Cancer Predictive Biomarker of Anti PD-1/PD-L1 Efficacy

**DOI:** 10.3390/cancers12092418

**Published:** 2020-08-26

**Authors:** Elise Ballot, Sylvain Ladoire, Bertrand Routy, Caroline Truntzer, François Ghiringhelli

**Affiliations:** 1 Cancer Biology Transfer Platform, Georges-François Leclerc Cancer Center, 1 rue du Professeur Marion, F-21000 Dijon, France; eballot@cgfl.fr (E.B.); sladoire@cgfl.fr (S.L.); 2 Bioinformatic Core Facility Georges-François Leclerc Cancer Center, F-21000 Dijon, France; 3 Centre de Recherche INSERM LNC-UMR1231, F-21000 Dijon, France; 4 Department of Medical Oncology, Centre Georges-François Leclerc, F-21000 Dijon, France; 5 UFR Santé, University Bourgogne Franche-Comté, F-21000 Dijon, France; 6 Genetic and Immunology Medical Institute, F-21000 Dijon, France; 7 Centre de Recherche du Centre Hospitalier de l’Université de Montréal (CRCHUM), Montréal, QC H2X 0A9, Canada; bertrand.routy@umontreal.ca; 8 Department of Medicine, Hematology–Oncology Division, Centre Hospitalier de l’Université de Montréal (CHUM), Montréal, QC H2X 0A9, Canada

**Keywords:** biomarkers, immunotherapy, biostatistics, tumor-infiltrating lymphocytes

## Abstract

Tumor immune infiltrates are associated with tumor prognosis in many cancer types. However, their capacity to predict the efficacy of checkpoint inhibitors is poorly documented. We generate three signatures that evaluate in different ways these infiltrates: lymphoid- and myeloid-alone signatures, and a combined signature of both named the TIL (tumor-infiltrating lymphocyte) transcriptomic signature. We evaluate these signatures in The Cancer Genome Atlas Program (TCGA) Pan-Cancer cohort and four cohorts comprising patients with melanoma, lung, and head and neck cancer treated with anti-PD-1 or anti-CTLA-4 therapies. We observe using TCGA Pan-Cancer cohort that this TIL or lymphoid-alone signature accurately estimates prognosis in most cancer types and outperforms histological TIL evaluation or myeloid signature alone. Both TIL and lymphoid signatures are correlated with response rate to immunotherapy. Combining lymphoid signature or TIL with tumor mutational burden generates a score that is highly efficient in predicting response to immunotherapy. In different series of patients treated with checkpoint inhibitors for non-small cell lung cancer, head and neck cancer, and melanoma, we observed that TIL or lymphoid signature were associated with outcome. These data demonstrate that a simple TIL or lymphoid signature could be used as a Pan-Cancer prognostic and predictive biomarker to estimate patient survival under checkpoint inhibitors.

## 1. Introduction

The revolution brought about by the advent of checkpoint inhibitors has changed the face of oncology, moving from cancer cell-centric investigation to immunocentered studies. Innate and adaptive immune response may detect cancer cells. Dendritic cells engulf dying cancer cells and present antigens to T-cells, which in turn migrate into the tumor to fight against tumor growth. Thus, there is a clear rational underpinning of the hypothesis that high-grade in situ immune response may be associated with better tumor control [1,2]. Many studies suggest that immune infiltrates with high densities of CD3 and CD8 T cells are associated with better outcomes [3]. In the context of colorectal cancer, the assessment of CD3 and/or CD8 cells by immunohistochemistry yields a substantial added value on top of the clinical evaluation for prognostic assessment [4,5]. In addition, tumor-infiltrating lymphocytes (TIL) scored on hematoxylin and eosin slides (H&E) also represent a standard tool for predicting cancer prognosis in breast cancer [6,7].

In the setting of immunotherapy, the only approved biomarker remains Programmed death-ligand 1 (PD-L1) assessment using immunohistochemistry, but it remains perfectible. Tumor mutational burden and microsatellite instability are emerging biomarkers that seem to have predictive value in different cancers [8,9,10,11]. There is some recent literature about the predictive role of immune infiltrate. Some reports focus on interferon gamma (IFNγ)-related mRNA signatures, which are linked to immune infiltrate and associated with a better efficacy of checkpoint inhibitors [12,13,14]. Other reports have tested the role of immune infiltrate to predict the efficacy of immunotherapy in the context of lung cancer and melanoma [15,16,17,18,19], and Luen et al. propose recommendations on current methodologies of evaluation, and the clinical relevance of TIL in the context of breast cancer [20].

Using transcriptomic data from tumor bulk, software such as Cibersort, or Microenvironment Cell Populations-counter (MCP-counter), we can extrapolate the quantity of immune cells [21,22]. Cibersort estimation of CD8 contained in the tumor was suggested to be associated with the checkpoint response rate [23]. However, the methodology is complex and requires a whole transcriptome.

We recently generated a simple transcriptomic signature using 40 genes that could mirror TILs, which are called the RNA TIL score [24]. More precisely, this score was generated using four 10-gene signatures, each signature representing respectively lymphoid, myeloid, stromal and tumor cells. Each signature was quantified through a score based on 10 genes selected as characteristics of the corresponding cellular subtype. Then, the TIL score was defined as the ratio of the sum of lymphoid and myeloid scores to the total of the 4 cell-type scores. Thus, by construction, the TIL score comprises other cell types compared to lymphoid and myeloid score. This TIL signature predicted prognosis in breast cancer in a manner similar to TIL assessment using H&E staining. In the present paper, we aimed to generalize the role of the TIL score, determine its prognostic value in the Pan-Cancer TCGA cohorts, and evaluate its utility for the prediction of immunotherapy efficacy. Besides, we investigated if using a lower number of genes through lymphoid alone or myeloid alone signature could be sufficient as a prognostic marker and predictive marker of immunotherapy efficacy.

## 2. Results

### 2.1. Evaluation of the TIL Composition in the Pan-Cancer TCGA Cohort

**Figure 1:**
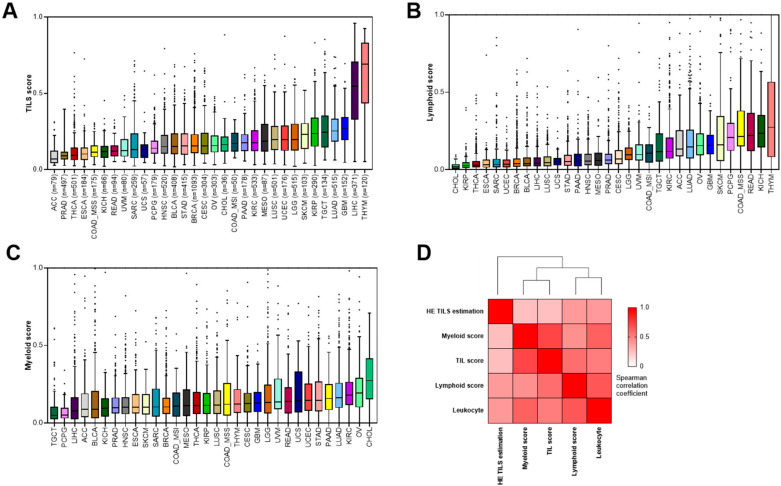
Evaluation of the tumor-infiltrating lymphocyte (TIL), lymphoid, and myeloid scores in the Pan-Cancer TCGA cohort. (**A**–**C**): Boxplots representing the distribution of the TIL (**A**), lymphoid (**B**), and myeloid (**C**) scores normalized between 0 and 1 according to tumor type for TCGA cohorts; for each score, tumor types are ordered by corresponding median values. (**D**): Spearman correlation matrix estimated for leukocyte, lymphoid, myeloid, TIL, and H&E TIL scores. Correlations were computed across all cancer types. H&E: hematoxylin and eosin.

The TIL score is composed of four 10-gene signatures that estimate lymphoid, myeloid, stromal, and tumor cell content from different cancer origins (see Methods and [24] for further details). The TIL score corresponds to lymphoid + myeloid score/total score, where the total score corresponds to lymphoid, myeloid, stromal, and tumor cells. In the present work, immune cell infiltrates were evaluated using our previously described TIL score and also using the individual lymphoid and myeloid 10-gene signature variables that composed the TIL score. The analysis was performed in the TCGA Pan-Cancer cohort, which includes 32 cancer types (Table S1) and 8975 patients. The TIL score varied substantially across tumor types (Figure 1A). The top 5 most infiltrated tumors were thymoma, liver hepatocellular cancer, glioblastoma, non-small cell lung cancer (NSCLC) adenocarcinoma, and testicular germ cell tumor. The 5 least infiltrated tumors were adrenocortical carcinoma, prostate adenocarcinoma, thyroid carcinoma, esophageal carcinoma, and colorectal cancer with microsatellite stable status. The top 5 tumors most invaded by lymphoid cells were thymoma, testicular germ cell tumor, clear cell kidney cancer, NSCLC adenocarcinoma, and cervical cell carcinoma (Figure 1B), while the top 5 tumors most invaded by myeloid cells were cholangiocarcinoma, ovarian cancer, kidney renal clear cancer, NSCLC, and pancreatic adenocarcinoma (Figure 1C).

Then, we tested the correlation between TIL score, lymphoid, and myeloid 10-gene signature variables, and performed a classical evaluation of leucocyte infiltration. As a gold standard, we used a histological estimation based on H&E diagnostic slides. The TCGA Pan-Cancer Atlas dataset includes representative H&E diagnostic whole-slide images that enable a spatial quantification and analysis of TIL, using a previously described artificial intelligence software [25]. This analysis is available for 13 cancer types and 3304 patients. We also used an additional classical variable described by the TCGA group, namely the leucocyte fraction using methylation analysis [26]. Matrix correlation between these variables showed significant albeit weak correlations between all these techniques, with Spearman correlation coefficients ranging from 0.26 to 0.72. H&E TIL estimation was more strongly correlated with lymphoid than with myeloid score (Figure 1D). Leucocyte fraction using methylation analysis was well correlated with lymphoid signature but weakly correlated with myeloid score.

Together, these data suggest that the TIL score is a valuable method to estimate both lymphoid and myeloid immune content in the TCGA Pan-Cancer cohort and suggests that H&E TIL estimation and leucocyte fraction using methylation analysis are more appropriate to study lymphoid accumulation, but not total immune infiltration.

### 2.2. Evaluation of the Immune Cells that Explain TIL, Lymphoid, and Myeloid Scores

**Figure 2:**
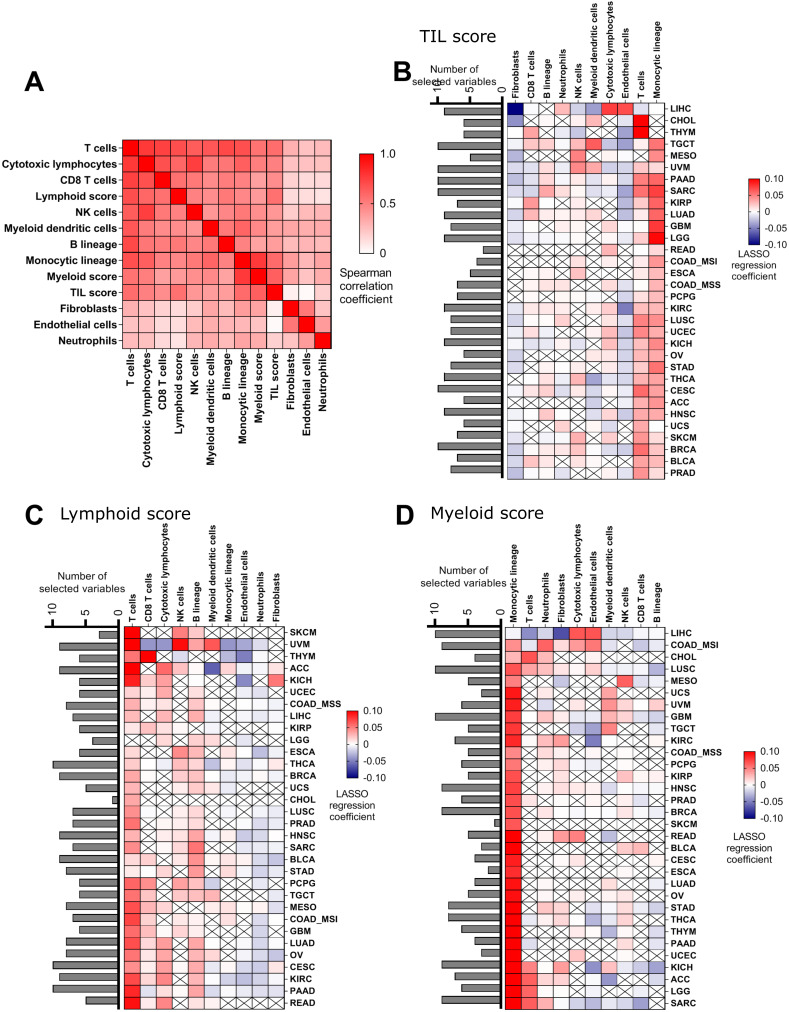
Relation between TIL, lymphoid, and myeloid scores respectively and MCP-counter abundances. (**A**): Spearman correlation matrix estimated for lymphoid, myeloid, and TIL scores as well as absolute abundances of eight immune and two stromal cell populations estimated by MCP-counter. (**B**–**D**): Heatmaps showing, for each cancer type, the regression coefficients estimated when fitting a LASSO model to describe the relationship between TIL score (**B**), lymphoid score (**C**), and myeloid score (**D**) and 10 cell population abundances. Bar plots in rows represent the number of variables selected in the LASSO model.

MCP-counter is a transcriptome-based computational method that quantifies the abundance of 8 major immune and 2 stromal cell populations from RNAseq tumor bulk [22]. The correlation matrix shows a strong correlation between all immune variables from MCP-counter and TIL score, except for neutrophils, and as expected, an absence of correlation with non-immune cells, fibroblast, and endothelial cells (Figure 2A). Similar correlations were observed between all MCP-counter variables and lymphoid or myeloid scores, except for neutrophil, fibroblast, and endothelial cells, thus suggesting a significant correlation between all immune variables in most cancer types.

Using MCP-counter, we assessed the capacity of TIL score to effectively represent the accumulation of lymphoid plus myeloid cells in the tumor bed. To identify variables associated with each score, we chose to use a Least Absolute Shrinkage and Selection Operator (LASSO) model. This model is a regression method that performs both variable selection and coefficient estimation. It allows selection of the minimal set of MCP-counter variables sufficient to predict each score. As to TIL score, results underline that in each cancer type, MCP-counter variables could predict the TIL content with good accuracy (mean squared error = 0.069), supporting our hypothesis that TIL effectively describes immune content. Across cancer types, a mean of 7 (range 5–10) MCP-counter variables were required to predict the TIL score (Figure 2B). In most cancer types, the two most important variables for predicting the TIL score are T lymphocytes and monocytes, which validates, using another methodological approach, the fact that the TIL score effectively represents the accumulation of lymphoid plus myeloid cells in the tumor bed. Using similar analysis, we validated the finding that only lymphoid and myeloid variables from MCP-counter respectively predicted lymphoid and myeloid scores (Figure 2C,D). Myeloid variables mainly reflect the accumulation of monocytes, while lymphoid variables mainly reflect T and B cells and to a lesser extent, Natural Killer (NK) and cytotoxic lymphocytes.

Together, these data underline that the TIL score is a good estimation of T lymphocyte and monocyte content in the TCGA Pan-Cancer cohort, while the lymphoid score signature is a good estimation of T, B, and (NK) cells, and the myeloid score is a good estimation of monocytes.

### 2.3. Evaluation of the Prognostic Role of the TIL Signature in the Pan-Cancer TCGA Cohort

**Figure 3:**
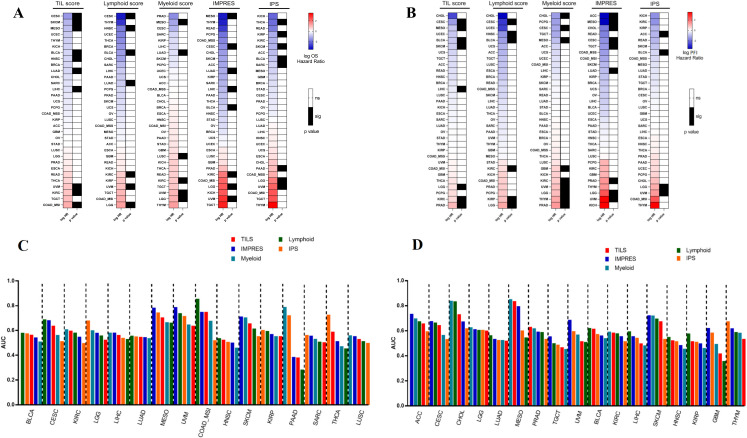
Evaluation of the prognostic role of the TIL and lymphoid scores in the Pan-Cancer TCGA cohort. (**A**,**B**): Log hazard ratios estimated by univariate Cox models for (**A**) overall survival (OS) and (**B**) progression-free interval (PFI) respectively associated with the TIL, lymphoid, myeloid, immunophenoscore (IPS) and IMmuno-PREdictive Score (IMPRES) scores, for each TCGA cancer type. Blue cells represent good prognosis and red cells represent poor prognosis. Black cells represent a *p* value for a log rank test less than 0.05. (**C**,**D**): For each significant TCGA tumor type from panel A and B respectively, bar plots are showing the area under the curve (AUC) estimated for overall survival (**C**) or progression-free interval (**D**) univariate survival models involving the five scores. For each tumor, the five scores are ordered decreasingly; the first score is the one that best predicts the outcome.

We next assessed the capacity of the TIL score and lymphoid or myeloid scores to predict prognosis in the Pan-Cancer TCGA cohort, and we compared their prognosis role to two signatures published as predictors of response to immune checkpoint blockade in melanoma, namely IMmuno-PREdictive Score (IMPRES) [27] and Immunophenoscore (IPS) [28]. IMPRES encompasses 15 pairwise transcriptomics relations between immune checkpoint genes. IPS is calculated as the sum of four scores corresponding to effector cells (activated CD4+ T cells, activated CD8+ T cells, effector memory CD4+ T cells, and effector memory CD8+ T cells), suppressive cells (regulatory T cells and myeloid-derived suppressor cells), Major Histocompatibility Complex (MHC)-related molecules, and checkpoints or immunomodulators. Using the TIL, lymphoid, and myeloid scores as continuous variables, we tested the capacity of these variables to predict OS (overall survival) and PFI (progression-free interval) (Figure 3A,B) through univariate Cox models. The TIL score was significantly associated with better OS and PFI for 7 and 5 cancer types, respectively. The lymphoid score was significantly associated with better OS and PFI for 4 and 3 cancer types, respectively. The myeloid score was significantly associated with OS and PFI for 2 and 3 cancer types, respectively. Interestingly, the TIL and lymphoid scores were associated with poor prognosis only in 2 and 3 cancer types (kidney, uveal melanoma, and colon cancer with microsatellite instability status for TIL and kidney and low-grade glioma for lymphoid score), while the myeloid score was associated with poor outcome in 4 cancer types (squamous lung, kidney, uveal cancer, and low-grade glioma). These results were very similar to IPS and IMPRES scores in terms of OS; our scores give notably better performance for head–neck squamous cell carcinoma or colon cancer with microsatellite instability status. The same observation was met for PFI. These results underline that our scores give equivalent and complementary information as previously published scores, using a limited number of genes.

Note that using dichotomic variables, the TIL score predicted OS and PFI in 11 and 14 cancer types, while the lymphoid score predicted OS and PFI for 14 and 16 cancer types (Table S2).

To go further, we then compared the performance of our scores to IMPRES and IPS for the prediction of OS and PFI using time-dependent area under the curve (AUC). The AUC of the TIL, lymphoid, and myeloid scores were very similar; they outperformed IMPRES and IPS in 9 cancers for the prediction of OS and 10 cancer types for PFI. IMPRES was the most predictive of only 3 and 6 cancer types, while IPS was the most predictive of 4 and 1 cancer types for the prediction of OS and PFI, respectively (Figure 3C,D).

To explore TIL and lymphoid scores further, we constructed a Cox model accounting for age, gender, tumor type, and tumor stage, which are hereafter defined as the “clinical only” model. Then, we constructed different models including clinical variables alone or adding the TIL score or H&E TIL to clinical variables. We also performed a similar analysis with clinical variables alone or adding the lymphoid score or H&E TIL (Tables S3 and S4). Taking OS as the outcome, the TIL score improved the accuracy of survival models in comparison to the clinical model in 9 cancer types. H&E TIL did not improve the accuracy of the model. In a similar analysis, the lymphoid score improved the accuracy of survival models in comparison to the clinical model in 6 cancer types, with no added value of H&E TIL.

Together, these data underline that the TIL score is a strong prognostic factor for OS and PFI in the Pan-Cancer TCGA cohort, highlighting the importance of the global estimation of the immune tumor microenvironment to estimate patient prognosis.

### 2.4. Evaluation of the TIL Score for the Prediction of Response to Anti PD-1/PD-L1 or CTLA-4

**Figure 4:**
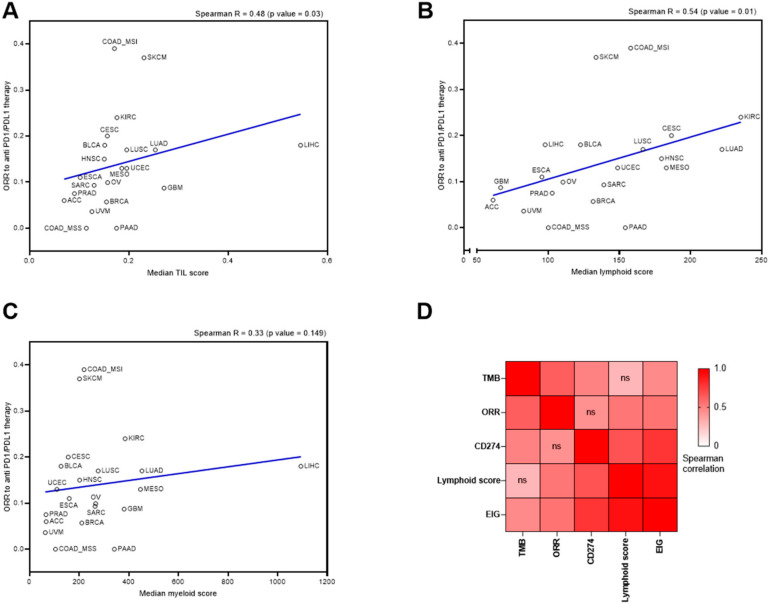
Evaluation of the predictive role of the TIL and lymphoid scores in the Pan-Cancer TCGA cohort. (**A**–**C**): Correlation of median TIL (**A**), myeloid (**B**), and lymphoid (**C**) scores with objective response rate (ORR) to anti-PD-1/PD-L1 therapy across 21 TCGA cancer types. (**D**): Spearman correlation matrix estimated for lymphoid score, TMB, CD274 gene expression, expanded immune gene (EIG) signature, and objective response rate (ORR) to anti-PD-1/PD-L1 therapy. Correlations were computed across 21 TCGA cancer types.

Then, we investigated whether the TIL, lymphoid, or myeloid scores could be surrogate biomarkers associated with clinical response to PD-1/PD-L1 inhibitors across multiple cancer types. To this end, we used the meta-analysis by Yarchoan et al. [29], which summarizes the objective response rate (ORR) to PD-1/PD-L1 inhibitor monotherapy in several published clinical trials. We assessed the correlation between the median TIL, lymphoid, and myeloid scores in the TCGA cohort in each cancer type and the ORR to PD-1/PD-L1 inhibitors (Figure 4A–C).

We observed that both the TIL and lymphoid scores were associated with ORR to PD-1/PD-L1 inhibitors; this was not the case for the myeloid score. The lymphoid score had a slightly higher correlation coefficient than the TIL score. We also performed a similar analysis with classical biomarkers, namely tumor mutational burden (TMB), extended immune signature (EIG) [12], and PD-L1 expression. As expected, all these variables were significantly correlated with ORR to PD-1/PD-L1 inhibitors (Figure S1A–C). We estimated a correlation matrix between TMB, EIG, PD-L1 expression (CD274 gene), and the lymphoid score, which was selected as a better new predictive variable than TIL (Figure 4D). While TMB was not correlated with EIG and lymphoid score, the EIG, PD-L1, and lymphoid score were strongly correlated (Figure 4D).

We performed multivariate linear regression including TMB, EIG, PDL1 expression, and the lymphoid score to estimate the model that best predicts ORR (Table 1).

Using the AIC, the best model for the prediction of ORR to PD-1/PD-L1 inhibitors combined TMB and the lymphoid score. Comparison using Anova of the models including TMB alone or TMB and the lymphoid score showed that the lymphoid score significantly improved the model (*p* = 0.05). Accordingly, the bivariate model comprising TMB and the lymphoid score showed a higher correlation with ORR than either TMB or the lymphoid score alone (Figure S1D).

Taken together, these data underline that the lymphoid score alone or in combination with PD-L1 or TMB could be a simple, general predictor of response to immune checkpoint inhibitors.

**Figure 5:**
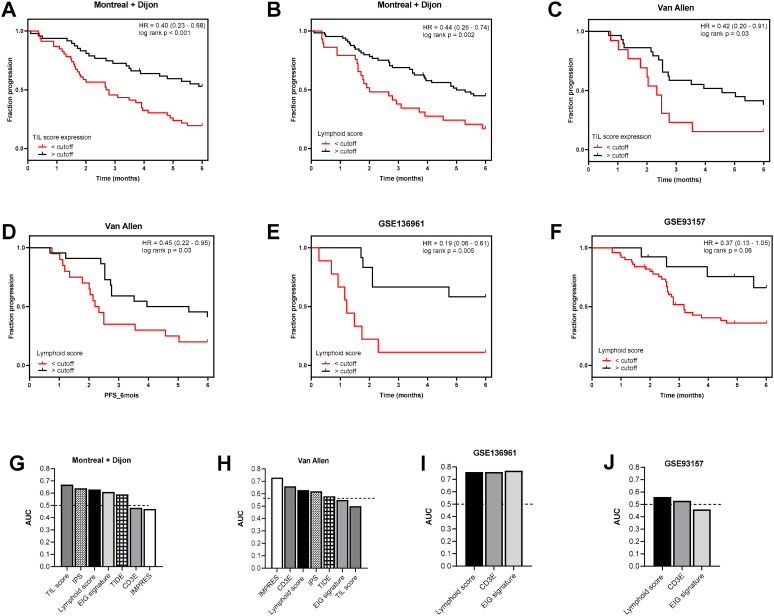
Prognostic role of TIL and lymphoid scores in comparison to other scores in a control cohort. (**A**–**F**): Kaplan–Meier survival analysis based on the TIL (**A**,**C**) and lymphoid (**B**,**D**–**F**) scores for progression-free survival; patients from the pooled Montreal and Dijon cohort (**A**,**B**), Van Allen (**C**,**D**), GSE136961 (**E**), and GSE93157 (**F**) were stratified according to the cutoff obtained from maximally selected rank statistics. (**G**–**J**): Bar plots showing area under the curve (AUC) estimated for progression-free interval in univariate survival models involving TIL, lymphoid, IMPRES, IPS, and Tumor Immune Dysfunction and Exclusion (TIDE) scores (when score was available), CD8A genes and EIG signature for pooled Montreal and Dijon (**G**), Van Allen (**H**), GSE136961 (**I**), and GSE93157 (**J**) cohorts.

Finally, to experimentally validate our results, we used different public or proprietary datasets of patients treated with anti PD-1/PD-L1 or CTLA-4. In the setting of NSCLC, we used a pooled analysis of 2 cohorts with lung cancer treated with atezolizumab, nivolumab, or pembrolizumab as monotherapy in Dijon (*n* = 43) and Montreal (*n* = 51) (Table S5). In addition, two public cohorts were used for anti PD-1/PD-L1. (1) The first was a cohort of 65 patients treated with nivolumab or pembrolizumab for melanoma, lung cancer, and head and neck cancer [30]; patients received pembrolizumab (57%) or nivolumab (43%) until progression or unacceptable toxicity. Patients with advanced melanoma could have received prior anti-CTLA4 therapy (7/28 patients). (2) The second was a cohort of 21 patients treated with nivolumab for lung cancer [31]. In our cohorts, we observed that high lymphoid and TIL scores were associated with better progression-free survival (Figure 5A,B). The same analysis was performed and results were validated for the Van Allen dataset in the context of metastatic melanoma and anti CTLA-4 therapy using ipilimumab antibody (Figure 5C,D). In the other two cohorts with anti PD-1/PD-L1, only a panel of genes was analyzed, and only the lymphoid score could be tested. In these two cohorts, a high lymphoid score was also associated with better outcome (Figure 5E,F).

In the 4 cohorts, using the AUC, we found that the lymphoid score or TIL score compared favorably to classical signatures such as EIG or immune infiltrate, as evaluated by CD3E expression (Figure 5G–J). In the Dijon–Montreal and Van Allen cohorts, TIL and lymphoid scores were also compared to IMPRES, Immunophenoscore (IPS), and TIDE (Figure 5G,H) [32]. TIDE is based on the modelization of the induction of T-cell dysfunction in tumors with high infiltration of cytotoxic T lymphocytes (CTL) and the prevention of T-cell infiltration in tumors with low CTL level; it provides a gene signature to model the tumor immune escape and was proposed as a surrogate biomarker to predict immune checkpoint blockade response. In the Dijon–Montreal cohort, the TIL score was more efficient than these 3 published scores. In the Van Allen cohort, where IMPRES was developed, the lymphoid score came just after IMPRES. Note that in the two last cohorts, all of the scores could not be tested due to many genes from the signature not being present in the dataset.

## 3. Discussion

TIL density is a strong positive prognostic indicator for many tumor types [3]. For example, an assessment of lymphocyte infiltration using known metrics such as the Immunoscore or our new software DGmuneS are strong predictors of OS in colorectal cancer [3,4,5,33,34]. The Immunoscore involves the quantification of CD8+ T cells at the center and periphery of a tumor, while the DGmuneS evaluates CD3 and tumor cells behavior. Likewise, H&E TIL assessment is a strong prognostic marker in localized breast cancer [6,7]. In the context of a checkpoint blockade, it has been shown that TIL density, as measured by immunohistochemistry (IHC) at the invasive margin of a tumor, as opposed to central infiltration, is most strongly associated with anti-PD-1 response [16]. In the setting of NSCLC, we also demonstrated that CD8 infiltration using IHC is a strong surrogate marker of a benefit of anti PD-1 monotherapy [15]. Although the approach consisting of TIL estimation using histology may be promising, standardization has been difficult, and additional data on the generalizability of this assay are needed. In particular, every study uses a different strategy, depending on the tumor type and the clinical setting. Thus, these results suggest that none of these biomarkers could be generalized in a Pan-Cancer analysis.

We recently generated a new transcriptomic signature called the RNA TIL score (20). This score was generated using four 10-gene signatures, representing respectively lymphoid, myeloid, stromal, and tumor cells. Each sample is described by 4 values, each summarizing one cellular subtype. The TIL score was defined as the ratio of the sum of lymphoid and myeloid scores to the total of the 4 cell-type scores. Using two large public datasets, we showed that a high RNA TIL score was significantly associated with the presence of a high level of TILs, as assessed by histology. The RNA TIL score was also associated with PFI (progression-free interval) and OS (overall survival) and was found to be an independent prognostic biomarker in triple-negative breast cancer [24].

In the present work, we first tested the prognostic role of TIL and lymphoid scores in a Pan-Cancer cohort. We observed that these scores were accurate biomarkers of PFI and OS in many cancer types used either as continuous or dichotomous variables. In most cancer types, the TIL score yielded similar results to the lymphoid score, and the lymphoid score could be a simpler surrogate marker of immune infiltrates. We observed some cancers for which a high TIL score was associated with worse outcome (kidney cancer and low-grade glioma). These findings are not surprising, because previous reports on IHC immune infiltrate underline that high CD8 content in such tumors is associated with poor outcome [35]. Interestingly, high myeloid infiltrate was associated with poor outcome in 5 cancer types, and it is only associated with good outcome in one cancer type. This observation suggests that myeloid cells mainly consist of immunosuppressive cells in some cancers [36], and consequently, extensive myeloid infiltration with modest lymphoid infiltration is a surrogate of poor prognosis. We hypothesize that on the basis of this information, cancers could be classified into three immunological types: some cancers for which immune response is associated with a state of immunosurveillance, other cancers for which immune response and especially myeloid response is associated with an immunosuppressive state that promotes tumor growth, and lastly, a group of cancers for which immune response is not involved in tumor prognosis and that are probably immune-ignored or immunotolerant. Together, these data support the rationale that an overall assessment of immune response using the TIL or lymphoid scores is sufficient to predict tumor prognosis.

In the context of checkpoint inhibitor blockade, lymphoid and TIL scores were accurate for predicting response to checkpoint inhibitors. These findings support the hypothesis that adaptive immunity has a positive role in inducing immune response after checkpoint inhibitor blockade and could be evaluated by these two simple biomarkers. Myeloid cells in the context of metastatic cancer may be enriched in immunosuppressive cells such as Type II tumor-associated macrophages or myeloid-derived suppressor cells, which could promote immunosuppression and blunt the efficacy of monoclonal antibody targeting checkpoint inhibitors [37]. This could temper the predictive value of the TIL score, explaining the slightly better efficacy of the lymphoid score to predict ORR to checkpoint inhibitors. Importantly, in the TCGA cohort correlation analysis, the lymphoid signature was improved by TMB assessment. A complete model that included TMB assessment in addition to the lymphoid score reduced the unexplained variance in predicting anti-PD-1/PD-L1 response rate across cancer types. Recently, three major papers have underlined that tertiary lymphoid structures and the accumulation of both B and T cells are key to the efficacy of checkpoint inhibitors [38,39,40]. As the lymphoid score accurately assesses B and T cell infiltration, we expect it might be a useful tool to estimate tumor infiltration in B and T cells or tertiary lymphoid structures. Importantly, in a local cohort using RNAseq, in 2 public cohorts using Nanostring technology or RNA panel sequencing, and in a public cohort of patients treated with anti CTLA-4, we validated the use of the lymphoid score to predict outcomes of patients treated with anti PD-1/PD-L1 as monotherapy for lung cancer, melanoma, or head and neck cancer. Our signature seems at least equivalent or better for some cohorts than previously published predictive signatures, which required large gene sets.

## 4. Materials and Methods

### 4.1. Patient Material

RNAseqV2 data with RSEM normalization of 31 cancer types from TCGA and corresponding clinical data were downloaded from the TCGA data portal. Tumor mutational burden (TMB) for each patient was downloaded from the National Cancer Institute [41]. Anti-PD-1/PD-L1 objective response rates (ORR) were available from Yarchoan et al. [29] for 21 cancer types. A gold-standard histological estimation based on H&E diagnostic slides was downloaded from Thorson et al. [26]. Four validation cohorts comprising patients treated with anti-PD-1 therapy were collected. The first is a private cohort including 94 patients with non-small cell lung cancer (NSCLC) for whom RNAseq data were generated in our NGS facility. The cohort was composed of 51 patients from Montreal and 43 patients from Dijon treated in first or second line by anti PD-1 or anti PD-1 as monotherapy. Clinical data were collected by Prof. F. Ghiringhelli and Dr B. Routy. Tumors were collected, stored, and used with the written informed consent of the patients. The study was performed in accordance with the Declaration of Helsinki.

The second cohort included 65 patients with melanoma, lung, and head and neck cancer for whom nCounter Pan-Cancer Immune Profiling Panel was performed. Expression data were generated using the nCounter system using the Pan-Cancer 730-Immune Panel [30]. The expression data profiling by array was downloaded from Gene Expression Omnibus (GEO) with GEO accession GSE93157.

The third cohort included data from 21 Chinese patients treated for NSCLC with anti PD-1 as monotherapy [31]. Expression data were generated using a panel of 395 immune-related genes with the Oncomine Immune Response Research Assay. The expression data profiling by array was downloaded from Gene Expression Omnibus (GEO) with GEO accession GSE136961.

A fourth validation cohort concerned 42 patients treated with anti-CTLA-4 therapy for whom RNAseq data were generated [42]. RPKM (Reads Per Kilobase Million) values were obtained from the authors.

### 4.2. RNA Sequencing Analysis for the Private Cohort

Total RNA was extracted from formalin-fixed paraffin-embedded (FFPE) tumor slices (5 × 5 µm) using the Maxwell 16 LEV RNA FFPE Purification kit (Promega) following the manufacturer’s instructions. Libraries were prepared from 12 µl of total RNA with the TruSeq Stranded Total RNA using Ribo-Zero (Illumina) following the manufacturer’s instructions. Once qualified, paired-end libraries were sequenced using 2 × 75 bp output on a NextSeq 500 device (Illumina). The abundance of transcripts from RNA-seq data was quantified through the Kallisto program. This program is based on pseudo alignment for rapidly determining the compatibility of reads with targets, without the need for alignment. The Kallisto transcript index used as a reference was built from merged human cDNA and ncDNA files from GRCh37 assembly ENSEMBL. Then, gene-level count matrices were created with the DESeq2 library. Low-count genes were pre-filtered by removing genes with too few reads.

### 4.3. Generation of TIL, Lymphoid, and Myeloid Scores and MCP-Counter Abundances

In a previous work, we generated a 40-gene signature specific to lymphocyte, myeloid, stromal, and cancer cells [24]. The 4 cell-type scores were computed by averaging the 10 selected probesets constituting the cell type for each sample. TIL score was defined as the ratio of the sum of lymphoid and myeloid scores to the total of lymphoid, myeloid, stromal, and cancer scores. In the present work, we focused on 3 scores, namely TIL, lymphoid, and myeloid.

MCP-counter abundances for 8 major immune and 2 stromal cell populations were estimated for each patient based on RSEM log2 transformed gene expressions and using “MCPcounter” R package [22].

### 4.4. Generation of IMPRES, Immunophenoscore (IPS), and TIDE Scores

IMmuno-PREdictive Score (IMPRES) scores were computed in each dataset using the expression function of 15 pairs of checkpoint genes as described by Auslander et al. [27]. The Immunophenoscore (IPS) was calculated on an arbitrary 0–10 scale based on the sum of the weighted averaged Z-score of the four following categories: effector cells (activated CD4+ T cells, activated CD8+ T cells, effector memory CD4+ T cells, and effector memory CD8+ T cells), suppressive cells (Tregs and MDSCs), MHC-related molecules, and checkpoints or immunomodulators [28]. Both scores were computed using codes provided by the authors. Tumor Immune Dysfunction and Exclusion (TIDE) scores were obtained through the web application provided by Jiang et al. [32].

### 4.5. Statistical Analyses

For each cancer type from TCGA, three LASSO regression models were fitted to predict each of our three scores, namely TIL, lymphoid, and myeloid scores, using MCP-counter estimated abundances as explanatory variables. Cox regression models were used to estimate hazard ratios (HR) and 95% confidence intervals (CIs) for overall survival (OS) and progression-free interval (PFI) or survival (PFS). A patient with an OS or a PFI time greater than 5 years and 3 years respectively was considered alive or without progression at this time. Survival curves were estimated by the Kaplan–Meier method and compared with the Log-rank test (univariate analysis). Optimal cutoffs for TIL and lymphoid scores were chosen based on maximally selected rank statistics [43]. The minimal and maximal proportions in groups were at least 0.2 and not more than 0.8 of the total observations.

Receiver Operating Characteristic (ROC) curve analysis was performed to evaluate the abilities of the different scores to predict prognosis. The time-dependent AUC (area under curve) values corresponding to the respective survival models were compared.

For a given cancer type, three nested Cox models were fitted to compare the effect of TIL, lymphoid, and H&E TIL scores in a clinical model. Clinical variables were age, sex, and tumor stage, when this latter was available. The comparison between the models without (clinical only) or with one of the three scores (full model) was performed by ANOVA.

All correlations were computed using nonparametric Spearman correlation.

Lymphoid and myeloid scores were scaled between 0 and 1 when only one given cancer type was considered.

Statistical tests were two-sided, and a *p*-value <0.05 was considered statistically significant. Data analysis was performed using R statistical software (http://www.R-project.org/) and presented with Prism 8 (GraphPad, San Diego, CA, USA).

## 5. Conclusions

In conclusion, we provide evidence that a 10-gene lymphoid transcriptomic signature could be used to predict response to immunotherapy. With the development of precision medicine, many cancers such as lung cancer require systematic RNAseq analysis, notably to address the presence of fusion that may be targeted by tyrosine kinase inhibitors. Adding 10 genes to this targeted RNAseq is a cost-effective strategy to provide additional predictive information regarding the capacity of the tumor to respond to checkpoint inhibitors alone. Our data strongly support the development of this strategy.

## Figures and Tables

**Table 1:** Summary of multivariate linear regression including TMB, EIG, PD-L1 expression, and lymphoid score to estimate the objective response rate (ORR). The best model was selected by the Akaike information criterion (AIC). TMB: tumor mutational burden; EIG: expanded immune gene signature.

	Full Model		Selected Model with AIC		Model with Only TMB	
	Coefficient (SE)	*p* Value	Coefficient (SE)	*p* Value	Coefficient (SE)	*p* Value
**(Intercept)**	0.041 (0.156)	0.795	0.011 (0.047)	0.827	0.099 (0.019)	5 × 10^−5^
**TMB**	0.011 (0.003)	0.002	0.01 (0.002)	0.001	0.011 (0.003)	4 × 10^−4^
**Lymphoid**	0.001 (0.001)	0.245	0.001 (0)	0.059	-	-
**CD274**	‒0.006 (0.023)	0.791	-	-	-	-
**EIG**	‒0.003 (0.031)	0.924	-	-	-	-
**R^2^**	0.584		0.581		0.486	
**Adjusted R^2^**	0.480		0.534		0.459	

## Supplementary Materials

The following are available online at https://www.mdpi.com/2072-6694/12/9/2418/s1, Figure S1: Evaluation of the predictive role of TMB score, CD274 expression, and EIG signature in the pan-cancer TCGA cohort, Table S1: TCGA Study Abbreviations, Table S2: Summary of univariate Cox models for overall survival (OS) and progression-free interval (PFI) respectively associated with the TIL, lymphoid and myeloid scores, for each TCGA cancer type, Table S3: Summary of likelihoods of different multivariate Cox models to predict the overall survival with TIL score, Table S4: Summary of likelihoods of different multivariate Cox models to predict the overall survival with Lymphoid score, Table S5: Summary of clinical characteristics of Montreal and Dijon cohorts.

## Code Availability

Code developed for the analysis is available upon request.
