# Supplementary material for: Tumor Infiltrating Lymphocytes Signature as a New Pan-Cancer Predictive Biomarker of Anti PD-1/PD-L1 Efficacy
Source: Cancers (Basel). 2020 Aug 26;12(9):2418. doi: 10.3390/cancers12092418 (PMC7564481; doi:10.3390/cancers12092418)
Supplement: Supplementary file 1 [file cancers-12-02418-s001.pdf]

cancers

MDPI

Supplementary Materials: Tumor Infiltrating Lymphocytes Signature as a New Pan-Cancer Predictive Biomarker of Anti PD-1/PD-L1 Efficacy

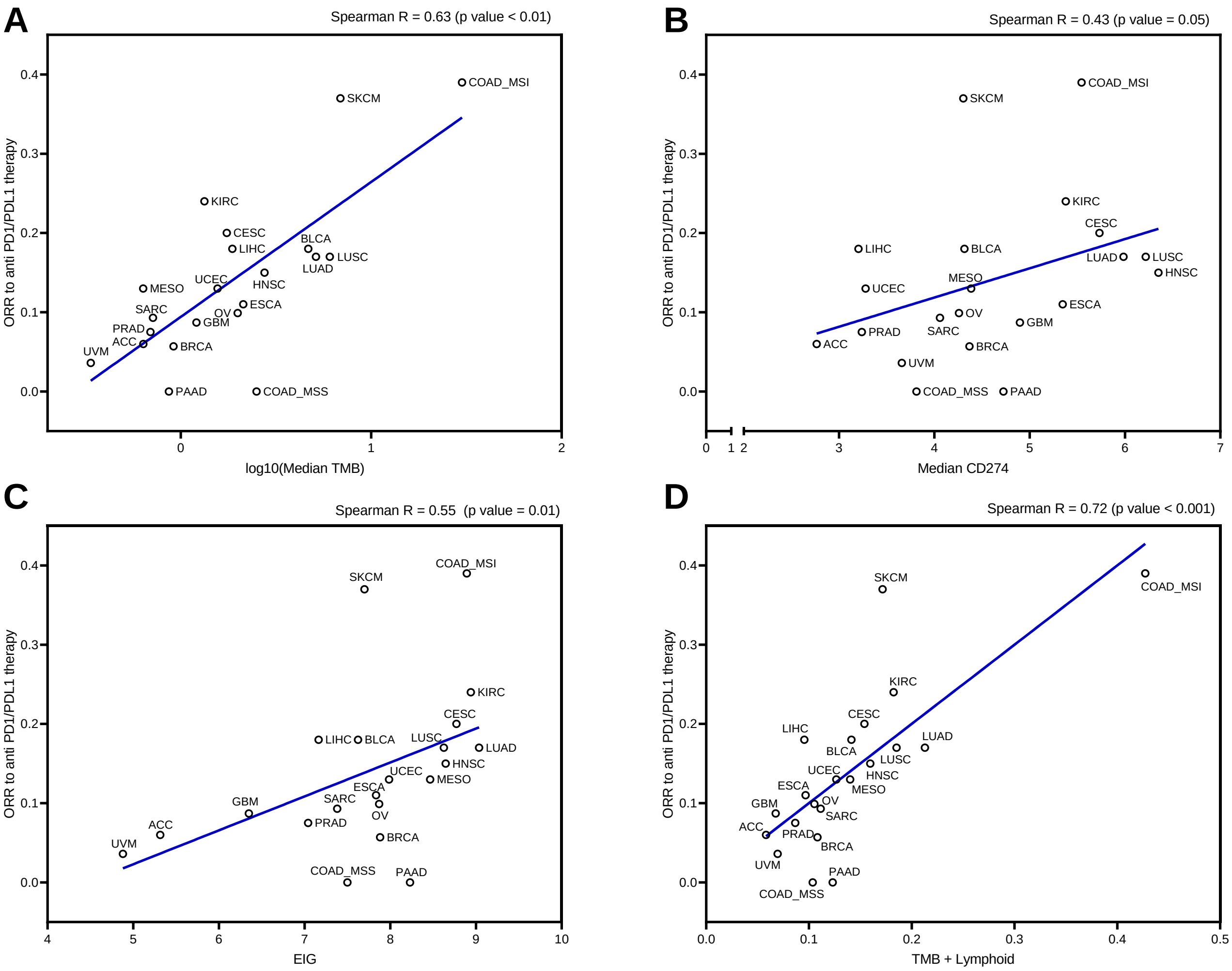

Figure S1. Evaluation of the predictive role of TMB score, CD274 expression and EIG signature in the pan-cancer TCGA cohort. **A**-**C**. Correlation of log10 median TMB (tumor mutational burden) (**A**), median of CD274 gene (**B**) and median EIG signature (**C**) with objective response rate (ORR) to anti-PD-1/PDL1 therapy across 21 TCGA cancer types. **D**. Bivariate model to predict the objective response rate (ORR) according to TMB (tumor mutational burden) and Lymphoid score. The regression formula for the objective response rate (ORR) is 0.011 + 0.01 x TMB + 0.001 x Lymphoid score.

Table S1. TCGA Study Abbreviations.

**ACC**	**Adrenocortical carcinoma**	**LUSC**	**Lung squamous cell carcinoma**
BLCA	Bladder Urothelial Carcinoma	MESO	Mesothelioma
BRCA	Breast invasive carcinoma	OV	Ovarian serous cystadenocarcinoma

CESC	Cervical squamous cell carcinoma and endocervical adenocarcinoma	PAAD	Pancreatic adenocarcinoma
CHOL	Cholangiocarcinoma	PCPG	Pheochromocytoma and Paraganglioma
COAD MSI	Colon adenocarcinoma with microsatellite instability status	PRAD	Prostate adenocarcinoma
COAD MSS	Colon adenocarcinoma with microsatellite stable status	READ	Rectum adenocarcinoma
ESCA	Esophageal carcinoma	SARC	Sarcoma
GBM	Glioblastoma multiforme	SKCM	Skin Cutaneous Melanoma
HNSC	Head and Neck squamous cell carcinoma	STAD	Stomach adenocarcinoma
KICH	Kidney Chromophobe	TGCT	Testicular Germ Cell Tumors
KIRC	Kidney renal clear cell carcinoma	THCA	Thyroid carcinoma
KIRP	Kidney renal papillary cell carcinoma	THYM	Thymoma
LGG	Brain Lower Grade Glioma	UCEC	Uterine Corpus Endometrial Carcinoma
LIHC	Liver hepatocellular carcinoma	UCS	Uterine Carcinosarcoma
LUAD	Lung adenocarcinoma	UVM	Uveal Melanoma

Table S2. Summary of univariate Cox models for overall survival (OS) and progression-free interval (PFI) respectively associated with the TIL, lymphoid and myeloid scores, for each TCGA cancer type. Patients were stratified according to the cutoff obtained from maximally selected rank statistics. L: low, H: high.

		**OS**				**PFI**			
		**TIL score**		**Lymphoid score**		**TIL score**		**Lymphoid score**	
		**HR [IC 95%]**	***p***	**HR [IC 95%]**	***p***	**HR [IC 95%]**	***p***	**HR [IC 95%]**	***p***
ACC	L	1		1		1		1	
	H	0,6 [0,24 ; 1,5]	0,27	0,46 [0,21 ; 1,03]	0,06	0,43 [0,22 ; 0,83]	**0,01**	0,31 [0,15 ; 0,65]	**0,00**
BLCA	L	1		1		1		1	
	H	0,64 [0,47 ; 0,86]	**0,00**	0,59 [0,43 ; 0,8]	**0,00**	0,58 [0,41 ; 0,81]	**0,00**	0,57 [0,41 ; 0,78]	**0,00**
BRCA	L	1		1		1		1	
	H	0,66 [0,41 ; 1,06]	0,08	0,56 [0,37 ; 0,84]	**0,00**	0,75 [0,47 ; 1,17]	0,20	0,65 [0,43 ; 1]	**0,05**
CESC	L	1		1		1		1	
	H	0,28 [0,14 ; 0,6]	**0,00**	0,29 [0,15 ; 0,54]	**0,00**	0,35 [0,19 ; 0,63]	**0,00**	0,37 [0,21 ; 0,65]	**0,00**
CHOL	L	1		1		1		1	
	H	0,37 [0,14 ; 0,98]	**0,05**	0,37 [0,14 ; 1,01]	0,05	0,31 [0,12 ; 0,81]	**0,02**	0,17 [0,06 ; 0,53]	**0,00**
COAD_MSI	L	-		1		1		1	
	H	-		7,7 [1,53 ; 38,85]	**0,01**	3,56 [0,89 ; 14,32]	0,07	4,21 [0,86 ; 20,51]	0,08
COAD_MSS	L	1		1		1		1	
	H	0,49 [0,24 ; 0,98]	**0,04**	0,52 [0,27 ; 1]	**0,05**	0,64 [0,35 ; 1,16]	0,14	0,53 [0,29 ; 0,97]	**0,04**

ESCA	L	1		1		1		1	
	H	1,8 [1,14 ; 2,85]	**0,01**	1,67 [1,03 ; 2,72]	**0,04**	1,5 [0,94 ; 2,4]	0,09	1,25 [0,82 ; 1,93]	0,30
GBM	L	1		1		1		1	
	H	0,73 [0,49 ; 1,09]	0,13	0,76 [0,52 ; 1,12]	0,17	1,62 [1,11 ; 2,36]	**0,01**	0,74 [0,51 ; 1,09]	0,13
HNSC	L	1		1		1		1	
	H	0,76 [0,57 ; 1,01]	0,06	0,65 [0,49 ; 0,86]	**0,00**	0,72 [0,53 ; 0,98]	**0,04**	0,62 [0,46 ; 0,84]	**0,00**
KICH	L	1		1		1		1	
	H	0,13 [0,02 ; 1,09]	0,06	1,99 [0,5 ; 7,96]	0,33	0,29 [0,06 ; 1,44]	0,13	4,25 [0,52 ; 34,58]	0,18
KIRC	L	1		1		1		1	
	H	1,9 [1,34 ; 2,69]	**0,00**	2,04 [1,37 ; 3,04]	**0,00**	1,83 [1,28 ; 2,62]	**0,00**	1,99 [1,3 ; 3,03]	**0,00**
KIRP	L	1		1		1		1	
	H	0,58 [0,27 ; 1,22]	0,15	2,87 [1,2 ; 6,86]	**0,02**	1,69 [0,86 ; 3,33]	0,13	2,49 [1,16 ; 5,33]	**0,02**
LGG	L	1		1		1		1	
	H	1,31 [0,86 ; 1,99]	0,20	1,76 [1,17 ; 2,67]	**0,01**	1,44 [1,05 ; 1,97]	**0,02**	1,27 [0,91 ; 1,78]	0,16
LIHC	L	1		1		1		1	
	H	0,65 [0,45 ; 0,94]	**0,02**	0,6 [0,4 ; 0,9]	**0,01**	0,65 [0,48 ; 0,89]	**0,01**	0,54 [0,38 ; 0,77]	**0,00**
LUAD	L	1		1		1		1	
	H	0,64 [0,47 ; 0,86]	**0,00**	0,59 [0,44 ; 0,79]	**0,00**	0,84 [0,62 ; 1,14]	0,26	0,58 [0,43 ; 0,78]	**0,00**
LUSC	L	1		1		1		1	
	H	1,31 [0,98 ; 1,76]	0,07	0,74 [0,55 ; 1]	0,05	0,78 [0,53 ; 1,13]	0,18	0,71 [0,5 ; 1,01]	0,06
MESO	L	1		1		1		1	
	H	0,42 [0,26 ; 0,69]	**0,00**	0,6 [0,37 ; 0,99]	**0,04**	0,4 [0,21 ; 0,75]	**0,00**	0,48 [0,28 ; 0,82]	**0,01**
OV	L	1		1		1		1	
	H	0,86 [0,63 ; 1,18]	0,36	0,73 [0,53 ; 1]	0,05	0,87 [0,65 ; 1,18]	0,39	0,79 [0,59 ; 1,06]	0,12
PAAD	L	1		1		1		1	
	H	0,76 [0,5 ; 1,15]	0,20	0,75 [0,46 ; 1,21]	0,24	1,31 [0,87 ; 1,97]	0,20	1,26 [0,85 ; 1,88]	0,24
PCPG	L	1		1		1		1	
	H	3,3 [0,55 ; 19,76]	0,19	0,55 [0,09 ; 3,27]	0,51	2,44 [0,91 ; 6,56]	0,08	3,04 [1,14 ; 8,15]	**0,03**
PRAD	L	1		1		1		1	
	H	3,56 [0,42 ; 30,51]	0,25	3,96 [0,73 ; 21,62]	0,11	2,43 [1,53 ; 3,87]	**0,00**	1,87 [1,18 ; 2,98]	**0,01**
READ	L	1		1		1		1	
	H	2,12 [0,79 ; 5,68]	0,13	0,64 [0,23 ; 1,74]	0,38	0,56 [0,21 ; 1,49]	0,24	0,38 [0,12 ; 1,18]	0,10
SARC	L	1		1		1		1	
	H	1,2 [0,78 ; 1,84]	0,41	0,75 [0,48 ; 1,18]	0,21	1,32 [0,92 ; 1,9]	0,13	1,21 [0,85 ; 1,71]	0,30
SKCM	L	1		1		1		1	
	H	0,37 [0,17 ; 0,8]	**0,01**	0,41 [0,19 ; 0,87]	**0,02**	0,36 [0,15 ; 0,86]	**0,02**	0,42 [0,21 ; 0,84]	**0,01**
STAD	L	1		1		1		1	
	H	1,27 [0,92 ; 1,74]	0,14	0,87 [0,63 ; 1,19]	0,38	0,89 [0,63 ; 1,26]	0,50	1,34 [0,93 ; 1,92]	0,11
TGCT	L	-		-		1		1	
	H	-		-		0,42 [0,16 ; 1,11]	0,08	0,31 [0,11 ; 0,88]	**0,03**
THCA	L	1		1		1		1	

	H	1,9 [0,53 ; 6,82]	0,32	1,63 [0,55 ; 4,88]	0,38	1,94 [1,03 ; 3,65]	**0,04**	1,41 [0,76 ; 2,63]	0,27
THYM	L	-		-		1		1	
	H	-		-		1,65 [0,56 ; 4,83]	0,36	0,55 [0,18 ; 1,74]	0,31
UCEC	L	1		1		1		1	
	H	0,52 [0,25 ; 1,07]	0,07	0,59 [0,28 ; 1,27]	0,18	0,47 [0,25 ; 0,89]	**0,02**	0,55 [0,26 ; 1,18]	0,12
UCS	L	1		1		1		1	
	H	0,48 [0,21 ; 1,11]	0,08	0,59 [0,28 ; 1,25]	0,17	0,64 [0,31 ; 1,3]	0,21	0,52 [0,26 ; 1,07]	0,08
UVM	L	1		1		1		1	
	H	2,66 [1,16 ; 6,07]	**0,02**	3,09 [0,92 ; 10,4]	0,07	2,01 [0,82 ; 4,94]	0,13	1,87 [0,63 ; 5,52]	0,26

Table S3. Summary of likelihoods of different multivariate Cox models to predict the overall survival. For the first analysis, the clinical model was compared to the clinical model with TIL score. The second analysis compared the two models including clinical information, TIL score and without and with H&E TIL score. H&E TIL score was available for only 13 cancer types.

	**Log-likelihood**		**F value**	***p***	**Log-likelihood**		**F value**	***p***
Model	Clinical model	Clinical model + TIL score	Anova (Df = 1)		Clinical model +TIL score	Clinical model + TIL and HE scores	Anova (Df = 1)	
ACC	-80.97	-80.94	0.07	0.79	–	–	–	–
BLCA	-895.64	-892.16	6.97	0.01	-610.83	-610.38	0.89	0.35
BRCA	-559.60	-558.81	1.59	0.21	-479.32	-479.23	0.19	0.67
CESC	-314.73	-307.10	15.25	0.00	-217.12	-215.47	3.30	0.07
CHOL	-49.27	-48.42	1.70	0.19	–	–	–	–
COAD MSI	-15.54	-13.11	4.85	0.03	-7.55	-7.55	0.00	0.97
COAD MSS	-143.26	-143.22	0.07	0.78	-130.29	-130.23	0.12	0.73
ESCA	-252.40	-252.26	0.29	0.59	–	–	–	–
GBM	-473.00	-472.87	0.26	0.61	–	–	–	–
HNSC	-960.78	-958.47	4.62	0.03	–	–	–	–
KICH	-20.47	-20.41	0.11	0.74	–	–	–	–
KIRC	-806.10	-804.80	2.61	0.11	–	–	–	–
KIRP	-144.29	-144.20	0.18	0.67	–	–	–	–
LGG	-507.64	-505.62	4.06	0.04	–	–	–	–
LIHC	-556.89	-555.32	3.13	0.08	–	–	–	–
LUAD	-893.21	-891.10	4.22	0.04	-773.16	-772.14	2.05	0.15
LUSC	-1000.21	-999.59	1.24	0.27	-592.13	-592.13	0.01	0.94
MESO	-252.62	-246.24	12.77	0.00	–	–	–	–
OV	-767.16	-767.06	0.19	0.66	–	–	–	–
PAAD	-401.45	-400.06	2.78	0.10	-370.04	-370.02	0.04	0.84
PCPG	-22.09	-21.99	0.21	0.65	–	–	–	–
READ	-37.55	-36.92	1,27	0.26	-30.97	-30.90	0.12	0.73
SARC	-431.21	-429.19	4.06	0.04	–	–	–	–

SKCM	-94.68	-92.53	4.30	0.04	-79.75	-79.72	0.05	0.82
STAD	-750.70	-750.34	0.71	0.40	-581.55	-581.32	0.44	0.51
TGCT	-12.81	-11.84	1.94	0.16	–	–	–	–
THCA	-55.91	-55.65	0.52	0.47	–	–	–	–
THYM	-25.21	-25.11	0.18	0.67	–	–	–	–
UCEC	-131.68	-131.27	0.82	0.36	-90.80	-90.26	1.07	0.30
UCS	-110.39	-110.21	0.35	0.55	–	–	–	–
UVM	-68.18	-66.61	3.14	0.08	-51.74	-51.51	0.45	0.50

Table S4. Summary of likelihoods of different multivariate Cox models to predict the overall survival. For the first analysis, the clinical model was compared to the clinical model with lymphoid score. The second analysis compared the two models including clinical information, lymphoid score and without and with H&E TIL score was available for only 13 cancer types.

	**Log-likelihood**		**F value**	***p***	**Log-likelihood**		**F value**	***p***
Model	Clinical model	Clinical model + lymphoid score	Anova (Df = 1)		Clinical model + lymphoid score	Clinical model + lymphoid and HE scores	Anova (Df = 1)	
ACC	-80.97	-80.77	0.41	0.52	-	-	-	-
BLCA	-895.64	-891.97	7.34	0.01	-610.99	-610.57	0.84	0.36
BRCA	-559.60	-558.63	1.94	0.16	-478.96	-478.81	0.29	0.59
CESC	-314.73	-306.12	17.22	0.00	-217.18	-215.31	3.73	0.05
CHOL	-49.27	-49.01	0.53	0.47	-	-	-	-
COAD MSI	-15.54	-13.75	3.57	0.06	-8.22	-8.11	0.24	0.63
COAD MSS	-143.26	-143.06	0.39	0.53	-130.33	-130.24	0.19	0.66
ESCA	-252.40	-252.39	0.02	0.89	-	-	-	-
GBM	-473.00	-472.72	0.57	0.45	-	-	-	-
HNSC	-960.78	-957.02	7.52	0.01	-	-	-	-
KICH	-20.47	-20.46	0.01	0.91	-	-	-	-
KIRC	-806.10	-805.69	0.83	0.36	-	-	-	-
KIRP	-144.29	-143.50	1.56	0.21	-	-	-	-
LGG	-507.64	-506.29	2.71	0.10	-	-	-	-
LIHC	-556.89	-556.72	0.33	0.57	-	-	-	-
LUAD	-893.21	-890.09	6.24	0.01	-772.37	-771.82	1.08	0.30
LUSC	-1000.21	-1000.18	0.06	0.81	-592.02	-591.99	0.07	0.79
MESO	-252.62	-252.44	0.35	0.55	-	-	-	-
OV	-767.16	-765.16	3.99	0.05	-	-	-	-
PAAD	-401.45	-399.91	3.08	0.08	-369.50	-369.48	0.04	0.85
PCPG	-22.09	-21.86	0.47	0.49			-	-
READ	-37.55	-37.45	0.20	0.66	-31.25	-31.15	0.20	0.66
SARC	-431.21	-429.56	3.31	0.07	-	-	-	-
SKCM	-94.68	-93.96	1.44	0.23	-80.95	-80.95	0.00	0.99
STAD	-750.70	-750.55	0.30	0.59	-582.09	-581.99	0.20	0.66
TGCT	-12.81	-12.14	1.33	0.25	-	-	-	-

THCA	-55.91	-55.26	1.30	0.25	-	-	-	-
THYM	-25.21	-22.83	4.75	0.03	-	-	-	-
UCEC	-131.68	-131.20	0.96	0.33	-90.64	-90.01	1.26	0.26
UCS	-110.39	-110.30	0.18	0.67	-	-	-	-
UVM	-68.18	-66.51	3.35	0.07	-52.00	-51.84	0.33	0.57

Table S5. Summary of clinical characteristics of Montreal and Dijon cohorts.

		**Montreal (*n* = 51)**	**Dijon (*n* = 43)**	**Total (*n* = 94)**
Age	Continuous (mean) [range]	66 [44 ; 83]	66 [46 ; 85]	66 [44 ; 85]
Sex	Female	27 (53%)	10 (33%)	37 (39%)
	Male	24 (47%)	33 (77%)	57 (61%)
Histology	Epidermoid	6 (12%)	20 (47%)	26 (28%)
	Adenocarcinoma	45 (88%)	22 (51%)	67 (71%)
	Other	0	1 (2%)	1 (1%)
Line	1	12 (24%)	2 (5%)	14 (15%)
	>1	39 (76%)	37 (86%)	76 (81%)
	NA	0	4 (9%)	4 (4%)
Treatment	Nivolumab	28 (55%)	42 (98%)	70 (74%)
	Pembrolizumab	17 (33%)	0	17 (18%)
	Other	6 (12%)	0	6 (6%)
	NA	0	1 (2%)	1 (1%)

© 2020 by the authors. Submitted for possible open access publication under the terms and conditions of the Creative Commons Attribution (CC BY) license (http://creativecommons.org/licenses/by/4.0/).
